# Bacterial Lysate from the Multi-Strain Probiotic SLAB51 Triggers Adaptative Responses to Hypoxia in Human Caco-2 Intestinal Epithelial Cells under Normoxic Conditions and Attenuates LPS-Induced Inflammatory Response

**DOI:** 10.3390/ijms24098134

**Published:** 2023-05-02

**Authors:** Francesca Lombardi, Francesca Rosaria Augello, Paola Palumbo, Laura Bonfili, Serena Artone, Serena Altamura, Jenna Marie Sheldon, Giovanni Latella, Maria Grazia Cifone, Anna Maria Eleuteri, Benedetta Cinque

**Affiliations:** 1 Department of Life, Health & Environmental Sciences, University of L’Aquila, 67100 L’Aquila, Italy; 2 School of Biosciences and Veterinary Medicine, University of Camerino, 62032 Camerino, Italy; 3 Dr. Kiran C Patel College of Osteopathic Medicine, Nova Southeastern University, Fort Lauderdale, FL 33314-7796, USA

**Keywords:** HIF-1α, PHD2, AKT, intestinal epithelial cells, probiotics, LPS

## Abstract

Hypoxia-inducible factor-1α (HIF-1α), a central player in maintaining gut-microbiota homeostasis, plays a pivotal role in inducing adaptive mechanisms to hypoxia and is negatively regulated by prolyl hydroxylase 2 (PHD2). HIF-1α is stabilized through PI3K/AKT signaling regardless of oxygen levels. Considering the crucial role of the HIF pathway in intestinal mucosal physiology and its relationships with gut microbiota, this study aimed to evaluate the ability of the lysate from the multi-strain probiotic formulation SLAB51 to affect the HIF pathway in a model of in vitro human intestinal epithelium (intestinal epithelial cells, IECs) and to protect from lipopolysaccharide (LPS) challenge. The exposure of IECs to SLAB51 lysate under normoxic conditions led to a dose-dependent increase in HIF-1α protein levels, which was associated with higher glycolytic metabolism and L-lactate production. Probiotic lysate significantly reduced PHD2 levels and HIF-1α hydroxylation, thus leading to HIF-1α stabilization. The ability of SLAB51 lysate to increase HIF-1α levels was also associated with the activation of the PI3K/AKT pathway and with the inhibition of NF-κB, nitric oxide synthase 2 (NOS2), and IL-1β increase elicited by LPS treatment. Our results suggest that the probiotic treatment, by stabilizing HIF-1α, can protect from an LPS-induced inflammatory response through a mechanism involving PI3K/AKT signaling.

## 1. Introduction

The hypoxia-inducible factor (HIF) pathway is a central and ubiquitous cellular mechanism promoting and coordinating the transcriptional response to low-oxygen (O_2_) environments [1]. In the intestine, several HIF target genes are involved in the maintenance of the mucosal barrier, including genes critical for microbial defense, xenobiotic clearance, mucin production, and cellular energetics [2]. HIFs are heterodimers consisting of a hypoxia-inducible α-subunit (HIF-1α, HIF-2α, and HIF-3α) and β-subunit (aryl hydrocarbon receptor nuclear translocator (ARNT)/HIF-1β and ARNT2) [3]. In normoxic conditions, HIF-α subunits are continuously synthesized while proteasomal pathways rapidly degrade them, limiting their circulating levels within cells [4]. Two prolyl residues in the O_2_-dependent degradation domain of HIF-α subunits are hydroxylated by the HIF prolyl 4-hydroxylases family (PHDs), primarily by PHD2 contributing to ubiquitination mediated by the von Hippel–Lindau (VHL) ubiquitin ligase [5,6]. Under hypoxia, when the cellular demand for O_2_ exceeds its supply, HIF-α subunits are stabilized following the loss of activity of PHDs due to the low availability of O_2_, one of their substrates. The subsequent translocation of the HIF-α subunit into the nucleus, where it dimerizes with HIF-1β, permits it to function as a transcription factor by binding to hypoxia-response elements of its target genes and leading to their expression [5,7].

The stabilization of HIF-1α associated with low O_2_ availability stimulates adaptive mechanisms to hypoxia, including enhancing anaerobic ATP generation through glycolysis [1]. Upregulated HIF-1α expression and increased glycolysis are dependent on the PI3K/AKT (protein kinase B) signaling, a pathway playing a central role in regulating energy metabolism. In particular, activated PI3K/AKT upregulates HIF-1α transcription and translation, stabilizing and trans-activating HIF-1α regardless of O_2_ levels [8,9]. In animal models, both genetic and pharmacologic stabilization of HIFs is disease protective. At the same time, the loss of these factors could lead to detrimental effects such as an increased susceptibility to colitis associated with diminished levels of epithelial HIF-1α [10,11,12,13,14].

Hypoxia mimetic agents able to increase HIF-1α expression have been hypothesized as potentially able to exert a positive effect in pathological contexts [15,16], such as gut inflammation, whereby the HIF pathway is induced by pharmacologic PHD inhibitors (PHI) [5,17]. PHI such as dimethyloxalylglycine (DMOG), FG-4497, AKB-4924, TRC160334, or CG-598 have been reported to exert protective effects in experimental murine colitis [12,13,18,19,20]. Recently, the effect of PHI on improving colonic anastomotic healing in a mouse model has been described [21].

Lipopolysaccharide (LPS) plays an important pathogenic role in intestinal inflammation: through the interaction with Toll-like receptors 4 (TLR4) it can activate the NF-κB pathways [22] that represent master regulators of inflammatory gene expression [23]. The increase in intestinal permeability associated with intestinal inflammation leads to the translocation of LPS into the blood circulation, triggering the release of pro-inflammatory cytokines such as tumor necrosis factor-α (TNF-α), IL-1β, or IL-6 [23]. These cytokines contribute to amplifying the activation of NF-κB signaling pathways in epithelial cells, decreasing the expression of tight junction (TJ) proteins, such as ZO-1 and occludin, and further increasing intestinal permeability [24]. NF-κB activation has been shown to stabilize HIF-1α in hypoxia and in inflammation. On the other hand, HIF-1α has been shown to repress NF-κB in vivo and in vitro under inflammatory conditions. It seems, therefore, that HIF-1α acts through negative feedback to NF-κB to reduce the host inflammatory response by restricting NF-κB-dependent gene expression [25,26].

The activation of NF-κB is also known to induce the expression of nitric oxide synthase 2 (NOS2), which is responsible for the increased NO production at the site of inflammation [27]. In the intestinal epithelium, the upregulation of NOS2 and the consequent chronic release of NO at high concentrations have been associated with the pathogenesis of inflammatory bowel disease (IBD) [28], mainly through the generation of peroxynitrite [29]. Moreover, NO can regulate HIF-1α accumulation, HIF-1 activity, and HIF-1-dependent target gene expression. However, studies addressing the regulation of HIF-1 by NO revealed a complex and paradoxical picture. It appears that short-term exposure to NO stabilizes HIF-1α, while chronic exposure to NO destabilizes HIF-1α. Several mechanisms were found to contribute to this variable role of NO in regulating HIF-1. It has been shown that NO regulates HIF-1 by modulating the activity of the O_2_-sensor enzymes, PHDs, and factor inhibiting HIF-1 (FIH-1) [30].

SLAB51 is a probiotic mixture consisting of *Streptococcus thermophilus* (DSM 32245), *B. lactis* (DSM 32246), *B. lactis* (DSM 32247), *L. acidophilus* (DSM 32241), *L. helveticus* (DSM 32242), *L. paracasei* (DSM 32243), *L. plantarum* (DSM 32244), and *L. brevis* (DSM 27961), previously studied both in vitro and in vivo, in animal models and patients, on hypoxia-related conditions [31,32,33,34,35,36]. Bacterial species present within this multi-strain probiotic formulation constitute common inhabitants of the human gut, mostly ingested with food, especially dairy products [37,38]. Consolidated knowledge shows that within the human gut, microorganisms are variably distributed with respect to quantity and types along the different gut districts. Environmental pH and available trophic sources represent the main factors driving such peculiar distribution, while age, geographical origin of the host, and lifestyle impact the global quantity of the different bacterial species in the gut microbiota [39,40]. Species of lactobacilli, streptococci, and bifidobacteria, present in the formulation used in our study, are mainly harbored in the most proximal parts of the gut such as the duodenum, jejunum, and ileum where a more limited bacterial load normally exists.

Several studies have registered beneficial effects of the multi-strain probiotic formulation, SLAB51, on humans and animal models; in particular, this probiotic proved to be protective in neurodegenerative disorders by promoting antioxidant and neuroprotective effects [31,41,42,43]. Moreover, it was able to keep the gut integrity, preserving its functionality in a mouse model of chemotherapy-induced peripheral neuropathy [44]. A recent study reported that SLAB51 alleviated the respiratory conditions in subjects with SARS-CoV-2 infections concluding that the probiotic was able to increase oxygen supply to other organs [33]. Of note, evaluating the neuroprotective effects of SLAB51 oral supplementation on neuroinflammation in Alzheimer’s disease (AD), a recent study showed its ability to induce HIF-1α stabilization and to reduce the PHD2 expression, along with inhibition of NOS2 expression and activity in the brain of an AD animal model [31].

Considering the crucial role of the HIF pathway in intestinal mucosal pathophysiology, we aimed to evaluate the ability of the SLAB51 probiotic lysate to influence the HIF-1α pathway and counteract the inflammatory stimulus of LPS in a model of in vitro intestinal epithelium.

Our findings show evidence that probiotic exposure, by positively impacting the HIF pathway through a mechanism involving PI3K/AKT upregulation, could predispose cells for better survival and reactivity in a pathologically hypoxic and inflammatory environment.

## 2. Results

### 2.1. Effect of SLAB51 Lysate on HIF-1α Levels on Caco-2 Cells

**Figure 1:**
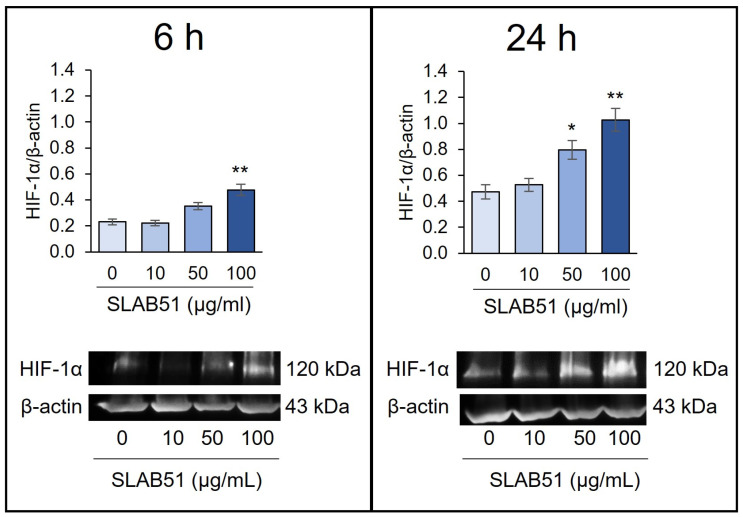
Effect of SLAB51 lysate on HIF-1α levels in Caco-2 cells. Cells were incubated with increasing concentrations of SLAB51 bacterial lysate for 6 and 24 h; HIF-1α levels were then evaluated by Western blotting. Following the densitometric analysis, the obtained values were normalized to β-actin. Values are expressed as means ± SEM of three independent experiments. Representative immunoblots are also shown. For the comparative analysis of the data, the one-way analysis of variance (ANOVA) followed by the Dunnett test was used. * *p* <0.05, ** *p* < 0.01 vs. untreated cells.

We first investigated the ability of SLAB51 to modulate HIF-1α levels on Caco-2 cells treated for 6 or 24 h with increasing concentrations of the probiotic lysate (10, 50, and 100 µg/mL). The treatment induced a dose-dependent increase in HIF-1α levels as assayed by Western blotting, being statistically significant after 6 h at 100 µg/mL (*p* < 0.01) (Figure 1). After 24 h of treatment, HIF-1α levels were significantly higher than the control at 50 and 100 µg/mL (*p* < 0.05 and *p* < 0.01, respectively, vs. untreated cells). Based on the obtained results, the SLAB51 concentration of 100 µg/mL, being the most efficient in influencing HIF-1α levels, was chosen for the following experiments.

### 2.2. Effect of SLAB51 Lysate on PHD2 Activity and Expression

**Figure 2:**
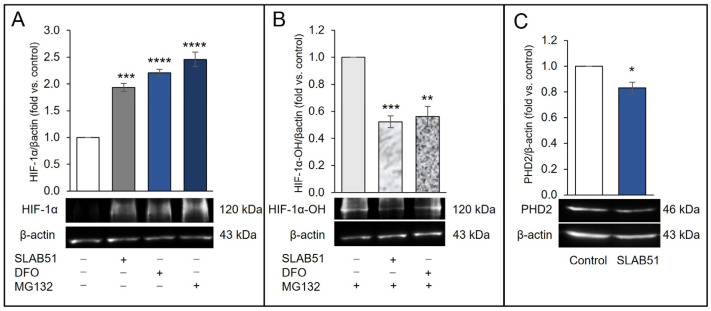
Effect of SLAB51 lysate on HIF-1α levels and PHD2 activity and expression. Cells were incubated in normoxia with SLAB51 lysate (100 µg/mL) or DFO (150 µM, positive control) with or without proteasome inhibitor MG132 (10 µM) for 6 h: (**A**) HIF-1α, (**B**) HIF-1α-OH, and (**C**) PHD2 levels were then evaluated by Western blotting. Following the densitometric analysis, the obtained values were normalized to β-actin. Values are expressed as means ± SEM of three independent experiments. Representative immunoblots are also shown. For the comparative analysis of the data, ANOVA followed by the Dunnett test or Student’s unpaired *t*-test were used, for comparison of the mean values among the groups and for comparison between two means, respectively. * *p* < 0.05, ** *p* < 0.01, *** *p* < 0.001, **** *p* < 0.0001 vs. untreated cells.

To investigate whether SLAB51 lysate affected HIF-1α levels through regulation of PHD2 activity or expression under normoxia, the levels of HIF-1α and hydroxylated HIF-1α were first analyzed. HIF-1α proline hydroxylation was determined using an antibody specific for the proline-hydroxylation site (Pro564) following proteasomal degradation inhibition through MG132 incubation to allow the accumulation of HIF-1α hydroxylated form. Immunoblotting in Figure 2 shows HIF-1α and hydroxyl-HIF-1α (HIF-1α-OH) protein levels. The effect of MG132 on proteasomal degradation inhibition of hydroxylated HIF-1α was demonstrated by the accumulation of HIF-1α (Figure 2A) as well as its hydroxylated form (Figure 2B). Treatment with deferoxamine (DFO) was used as a positive control, being an iron chelator known to attenuate PHD activity and, in turn, stabilize HIF-1α; it increased HIF-1α and reduced the accumulation of (hydroxyproline) Hyp-564 under normoxia (Figure 2B). Likewise, SLAB51 treatment decreased Hyp-564 compared with the control under normoxia after 6 h of incubation, increasing HIF-1α levels. We next assessed the ability of SLAB51 lysate to influence PHD2 expression. The treatment with the probiotic lysate significantly reduced PHD2 expression at 6 h (Figure 2C) as evaluated through Western blot (% reduction vs. control ~17%).

### 2.3. Effect of SLAB51 Lysate on Cell Metabolism and AKT Pathway

**Figure 3:**
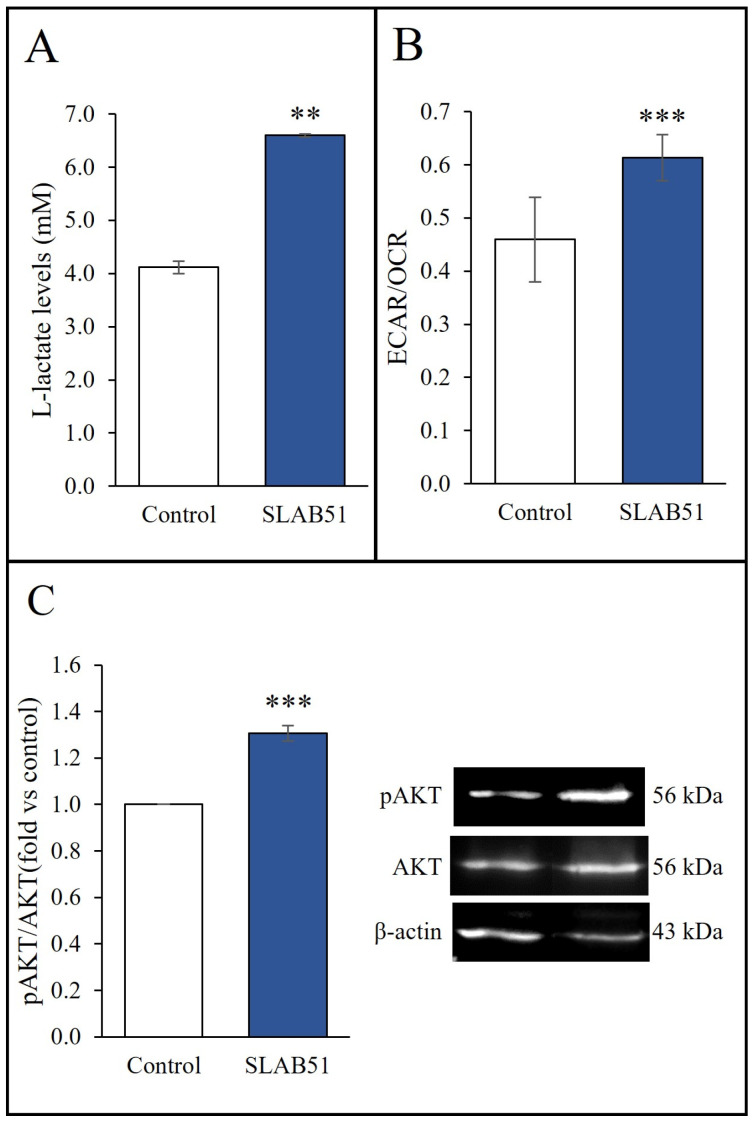
Effect of SLAB51 lysate on L-lactate production, glycolysis rate, and pAKT levels. Caco-2 cells were treated with or without SLAB51 lysate (100 µg/mL) for (**C**) 6 h or (**A**,**B**) 24 h. The levels of lactate were analyzed through a colorimetric assay on cell culture supernatants. Values are expressed as the means ± SEM of three independent experiments in triplicate (**A**). The relative glycolysis rate ECAR/OCR was assessed using the Seahorse XF Analyzer. Values are expressed as the means ± SD of a representative experiment (N = 9) out of three independent experiments (**B**). pAKT levels were evaluated by Western blotting. Following the densitometric analysis, the obtained values were normalized vs. AKT. Values are expressed as the means ± SEM of three independent experiments. Representative immunoblots of pAKT, AKT, and β-actin are also shown (**C**). For comparison between two means, the Student’s unpaired *t*-test was used ** *p* < 0.01; *** *p* < 0.001.

Given the ability of HIF-1α to induce genes encoding glycolytic enzymes [45], the effect of SLAB51 on glycolytic metabolism was studied next. The levels of L-lactate, a key metabolite of the glycolysis pathway, were evaluated in culture media. The relative glycolysis rate was also analyzed as the ECAR/OCR ratio. ECAR stands for the extracellular acidification rate of the media, a parameter that reflects glycolysis, while OCR is the O_2_ consumption rate used to determine oxidative phosphorylation [46]. Both levels of L-lactate (Figure 3A) and the ECAR/OCR ratio were significantly increased in Caco-2 cells after 24 h incubation with the probiotic lysate (Figure 3B). Upregulated HIF-1α expression and increased glycolysis are dependent on the activated PI3K/AKT pathway, which plays a central role in regulating the energy metabolism [8]. To verify the ability of SLAB51 lysate to modulate the AKT pathway, the analysis of AKT phosphorylation was performed. As shown in Figure 3C, phospho-AKT (pAKT, Ser473) protein levels were significantly upregulated after 6 h treatment with SLAB51 lysate compared with control cells.

### 2.4. Involvement of AKT Pathway in SLAB51′s Ability to Increase HIF-1α Levels

**Figure 4:**
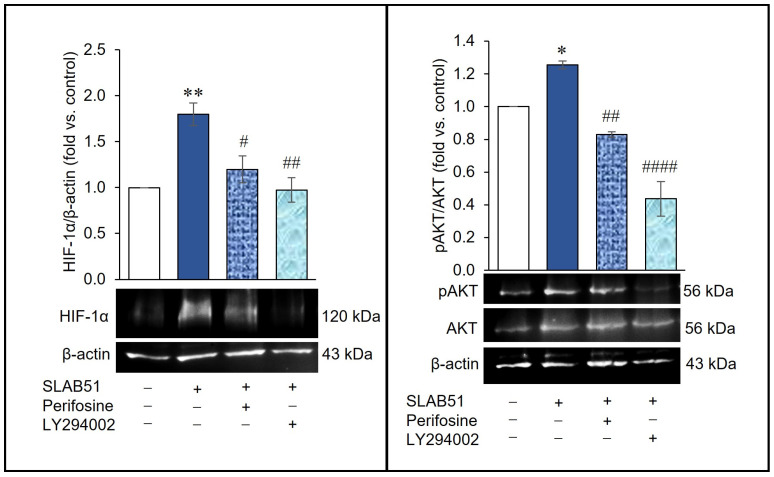
Effect of AKT and PI3K inhibition on SLAB51 lysate’s ability to increase HIF-1α levels. Caco-2 cells were pre-treated with perifosine or LY294002 for 30 min and then incubated with SLAB51 lysate (100 µg/mL) for 6 h. HIF-1α and pAKT levels were evaluated by Western blotting. Following the densitometric analysis, the obtained values were normalized to β-actin or AKT. Representative immunoblots of HIF-1α, pAKT, AKT, and β-actin are also shown. Values are expressed as the means ± SEM of three independent experiments. The ANOVA followed by the Tukey test was used for the comparative analysis of the data. * *p* < 0.05, ** *p* < 0.01 vs. untreated cells; # *p* < 0.05, ## *p* < 0.01, #### *p* < 0.0001 vs. SLAB51.

The involvement of the PI3K/AKT pathway was further verified by analyzing the influence of perifosine and LY294002, i.e., AKT and PI3K inhibitors, respectively, on the effect of SLAB51 on HIF-1α levels of Caco-2 cells. The cells were pre-treated for 30 min with perifosine or LY294002 and then incubated with SLAB51 lysate for 6 h. Pretreatment with each inhibitor counteracting the pAKT increase induced by SLAB51 inhibited its ability to increase HIF-1α, lowering the protein expression to levels comparable to those of the control (Figure 4). These data suggest that the activation of the PI3K/AKT pathway is required for the HIF-1α increase induced by SLAB51 lysate treatment.

### 2.5. Effect of SLAB51 Lysate on HIF-1α Levels on an In Vitro Model of Intestinal Inflammation

**Figure 5:**
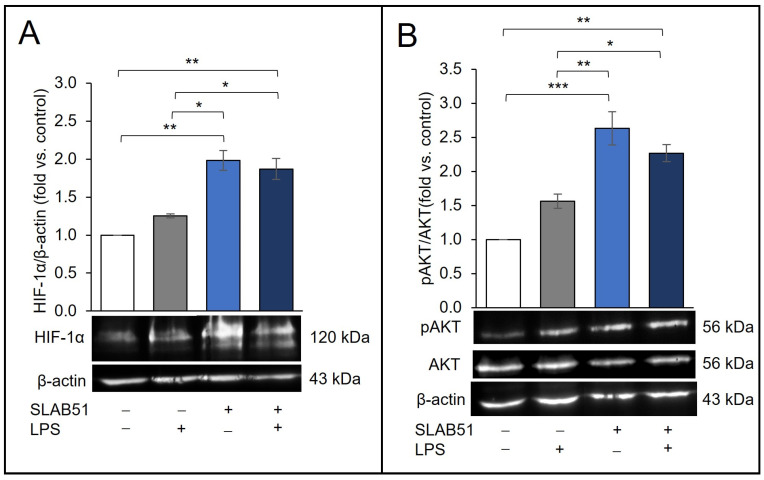
Effect of SLAB51 lysate on HIF-1α and pAKT levels in LPS-stimulated Caco-2 cells. Caco-2 cells were treated for 24 h with or without LPS (10 µg/mL) and SLAB51 lysate (100 µg/mL). (**A**) HIF-1α and (**B**) pAKT levels were evaluated by Western blotting. Following the densitometric analysis, the obtained values were normalized with respect to β-actin or AKT. Values are expressed as the means ± SEM of three independent experiments. Representative immunoblots are also shown. For the comparative analysis of the data, ANOVA was used followed by the Tukey test. * *p* < 0.05, ** *p* < 0.01, *** *p* < 0.001,.

The effect of the SLAB51 lysate was then evaluated on Caco-2 cells exposed to LPS, 10 µg/mL. In our experimental conditions at 24 h, LPS treatment was unable to increase HIF-1α levels, which were not different compared to the control. On the other hand, the ability of the SLAB51 treatment to increase HIF-1α levels was not affected by the concomitant treatment with LPS (Figure 5A). The effect on AKT activation was also evaluated. LPS did not significantly influence the AKT pathway nor the ability of SLAB51 lysate to increase pAKT levels (Figure 5B).

### 2.6. Probiotic Prevention of LPS-Induced Pro-Inflammatory Stress in Caco-2 Cells

**Figure 6:**
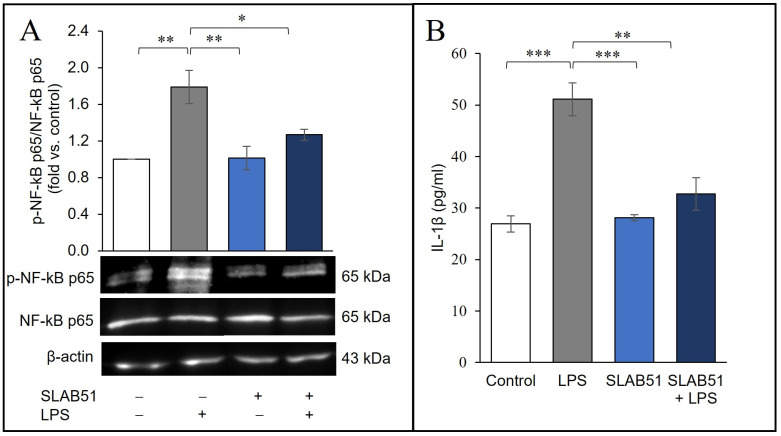
Effect of SLAB51 lysate treatment on inflammatory markers in LPS-stimulated Caco-2 cells. Caco-2 cells were treated for 24 h with or without LPS (10 µg/mL) and SLAB51 lysate (100 µg/mL). (**A**) p-NF-κB p65 and NF-κB p65 were analyzed by Western blotting. Following the densitometric analysis, the obtained values for p-NF-κB p65 were normalized with respect to NF-κB p65. Representative immunoblots of p-NF-κB p65, NF-κB p65, and β-actin are also shown. (**B**) IL-1β levels in the culture medium were assayed by ELISA kit. Data shown are expressed as mean ± SEM of three independent experiments. For the comparative analysis of the data, ANOVA was used followed by the Tukey test. * *p* < 0.05, ** *p* < 0.01, *** *p* < 0.001.

As known, LPS induces NF-κB signaling and upregulates the production of inflammatory cytokines at the intestinal level [23]. Moreover, in our experiments, LPS significantly enhanced the levels of phospho-NF-κB p65 in Caco-2 cells (Figure 6A) and the secretion of the pro-inflammatory cytokine IL-1β into the culture medium (Figure 6B). Of note, these effects were counteracted by the treatment with SLAB51 lysate.

**Figure 7:**
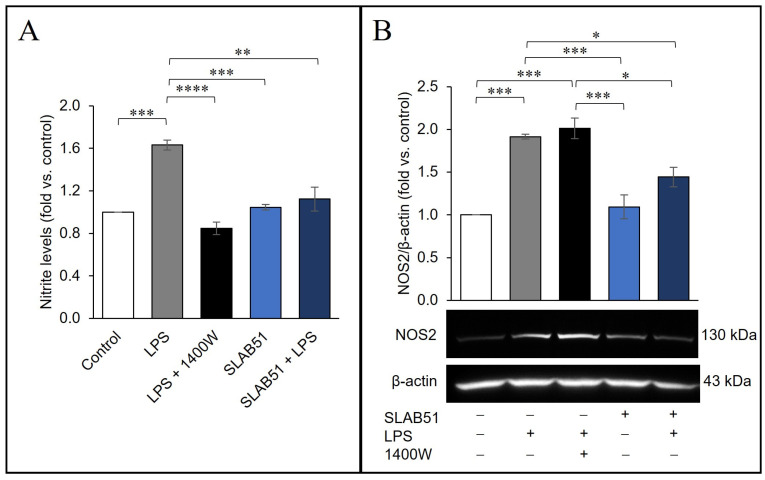
Effect of SLAB51 lysate on NOS2 expression and activity in LPS-stimulated Caco-2 cells. Caco-2 cells were treated for 24 h with or without LPS (10 µg/mL), NOS2 inhibitor 1400W (100 µM), SLAB51 lysate (100 µg/mL). (**A**) Nitrite levels in the culture medium were assayed by Griess reagent. Values are expressed as fold of nitrite levels vs. control. (**B**) Western blotting for NOS2 on cell lysates. Following the densitometric analysis, the obtained values were normalized with respect to β-actin. Representative immunoblots of NOS2 and β-actin are also shown. Data shown are expressed as means ± SEM of three independent experiments. For the comparative analysis of the data, ANOVA was used followed by the Tukey test. * *p* < 0.05, ** *p* < 0.01, *** *p* < 0.001, **** *p* < 0.0001.

The transcription factor NF-κB represents a key mediator of inflammatory responses as it is able to induce the expression of various pro-inflammatory genes, including NOS2 [47]. Thus, the effect of SLAB51 lysate was next assessed on the expression and activity of NOS2 induced by LPS. Nitrite levels in the culture medium at 24 h after LPS stimulation were increased with respect to control cells. The treatment with SLAB51 abrogated the ability of LPS to promote NOS2 activity, bringing the nitrite levels back to those of the control (Figure 7A); the specificity of the assay was verified using the NOS2 inhibitor, 1400W. In Figure 7B, the results of Western blot analysis of NOS2 protein are shown; as expected, LPS was able to induce the NOS2 expression. Of note, this effect was counteracted by the concomitant treatment with SLAB51 lysate, with the NOS2 expression not significantly different from untreated controls. As expected, no effect on NOS2 protein level was observed after treatment with the inhibitor of the NOS2 activity, 1400W.

**Figure 8:**
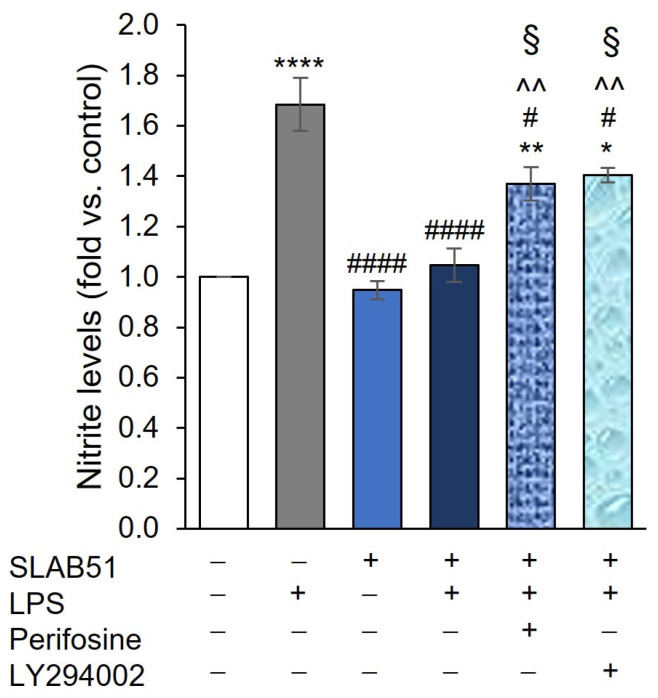
Effect of AKT and PI3K inhibition on SLAB51 lysate’s ability to modulate NOS2 activity in LPS-stimulated Caco-2 cells. Caco-2 cells were incubated in the presence or absence of perifosine or LY294002 for 30 min and then treated for 24 h with or without LPS (10 µg/mL) and SLAB51 lysate (100 µg/mL). Nitrite levels in the culture medium were assayed by Griess reagent. Values are expressed as fold of nitrite levels vs. control. Data shown are expressed as means ± SEM of three independent experiments. For the comparative analysis of the data, ANOVA was used followed by the Tukey test. * *p* < 0.05, ** *p* < 0.01, **** *p* < 0.0001 vs. control; # *p* < 0.05, #### *p* < 0.0001 vs. LPS; ^^ *p* < 0.01 vs. SLAB51; § *p* < 0.05 vs. SLAB51 + LPS.

Finally, we verified the hypothesis that the anti-inflammatory effect of SLAB51 observed in our model could be mediated by the ability of the probiotic to modulate the HIF-1α pathway. For this purpose, the cells were incubated with AKT and PI3K inhibitors 30 min prior to the treatment with SLAB51 lysate and LPS, and then the effect on NOS2 activity was evaluated after 24 h treatment. The results demonstrated that the two inhibitors were able to significantly interfere with the ability of SLAB51 lysate to reduce NOS2 activity (Figure 8). Indeed, nitrite levels in the culture medium after SLAB51 + LPS + perifosine or LY24002 were significantly higher than SLAB51 + LPS treatment (*p* < 0.05). However, both inhibitors were unable to totally abrogate the effect of SLAB51 since nitrite levels in the medium of the cells treated also with perifosine or LY24002 were still significantly lower than LPS treatment (*p* < 0.05).

## 3. Discussion

The role of the HIF pathway in regulating gut homeostasis is an area of active interest, as demonstrated by the plethora of studies highlighting the therapeutic potential of targeting pathological hypoxia signaling pathways in IBD [16,48]. Among HIFs, the HIF-1α subunit is the most ubiquitously expressed. Recent studies emphasize the potential role of HIF-1α as the central hypoxia sensor in preserving gut homeostasis by improving the survival of gut microresidents [2,3,49]. In addition, HIF-1α plays a crucial role in maintaining the integrity of the intestinal epithelial mucosa by upregulating genes involved in gut barrier functions such as MUC2, ITF, CLDN1, and other tight junction proteins [2].

Despite the increasing attention on the role of HIFs in regulating mucosal barrier function, metabolism, inflammatory processes, and immune response in the gut, the ability of probiotics to influence the HIF pathway has not been thoroughly investigated. The existing studies evaluating probiotics’ influence on HIF-1α stabilization in the intestinal environment are limited to investigating the effect of short-chain fatty acids, mainly butyrate, a product of microbial fermentation of dietary fibers in the lower intestinal tract able to directly and non-competitively inhibit PHD [50]. This effect has been linked to the colonization of the colon by probiotics while no evidence has been produced about activities directly exerted by probiotics in their transit through the upper intestine.

Ingested probiotics must pass the stomach, survive the gastric acid, bile salts, and pancreatic enzymes, and travel through the intestine until they reach the ileocecal valve. It is the common opinion that a substantial reduction in viable probiotic bacteria during transit in the gastrointestinal tract reduces the benefits of probiotics [51]. Even though the joint FAO/WHO working group specifically recommended that candidate bacteria for use as probiotics be resistant to gastric acidity and bile compounds, there is always a proportion of bacteria undergoing destruction before reaching the colon. The contents of these destroyed bacteria could have a biological effect at the small intestine level, where the bulk of nutrient and calorie absorption takes place. So, even though the number of bacteria in the proximal tract of the intestine is low, their biological relevance may play a pivotal role in influencing the host physiology as many of the systemic effects of the gut are generated in the small intestine [52]. Particularly, bacteria in the duodenum and the upper part of the intestine could influence hypoxia through the modulation of the HIF pathway. It has been recently reported that vertical sleeve gastrectomy (VSG) facilitated the richness of Lactobacillus spp. by reducing the amount of gastric acid reaching the duodenum and was associated with a consistent increase in HIF2α signaling [53].

Regarding the possibility of a direct action of the probiotic at the level of the duodenum and upper part of the intestine, we would like to mention a recent study by Ceccarelli et al., which has reported a significant and rapid improvement in the respiratory conditions of patients positive for SARS-CoV-2 infection if treated with SLAB51 [33]. After 24 h from the start of bacteriotherapy, the group treated with SLAB51 had an improved blood oxygenation compared to the group that received only routinely administered anti-COVID-19 treatment, as evidenced by the analysis of pO_2_, O_2_Hb, and SaO_2_ values. The mechanism by which SLAB51 lysate leads to the improvement of lung functions are not yet identified. Furthermore, treatment with SLAB51 has recently been shown to induce HIF-1α stabilization, reduce PHD2 expression, and inhibit NOS2 expression in the brain of the AD mouse model [31]. These findings indicate that the probiotic formulation can exert positive effects not only in the gastrointestinal tract, but also in extraintestinal organs. In the small intestine, the modulation of the HIF pathways is more probably a consequence of direct, enzymatic action of the probiotic rather than the result of the bacterial fermentation process, as could be in the colon.

Driven by the need to deepen our understanding of the crucial role of the HIF in intestinal mucosal physiology, in this study, we have evaluated the effect of the SLAB51 lysate on the HIF pathway in Caco-2 IECs. The data obtained show that exposure of Caco-2 IECs to SLAB51 under normoxic conditions positively influenced the HIF pathway. Specifically, a dose-dependent upregulation in HIF-1α levels was associated with an increase in L-lactate levels and the relative glycolysis rate in the treated cells. SLAB51′s ability to stabilize HIF-1α was associated with the inhibition of PHD2 activity and expression. The involvement of the PI3K/AKT pathway underlying the ability of the SLAB51 lysate to induce the increase in HIF-1α levels was also verified, per previous reports supporting that upregulated HIF-1α expression and increased glycolysis are dependent on the activation of this pathway, regardless of oxygen levels [8]. Moreover, PHD2 inhibition induced by SLAB51 lysate could also lead to AKT activation, thus indirectly contributing to the observed increase in HIF-1α levels [54,55]. PHD2 can directly hydroxylate AKT on two major proline residues, thus leading to its inactivation [54]. On the other hand, the deletion of PHD2 leads to AKT activation [55]. Of note, the activation of PI3K/AKT signaling in melanoma cells has been reported to induce PHD2 inhibition, thereby promoting HIF-1α stability [56].

Although preliminary, our results are supported by previously published clinical data [31,32,33,34]. In this context, our observations lead us to hypothesize that the multi-strain probiotic SLAB51 is capable of conditioning cells to make them more prone to survive and respond to stresses to which they are exposed in a hypoxic and inflamed environment.

Probiotics have been shown to suppress inflammation by inhibiting various signaling pathways, such as the NF-κB pathway [57]. NF-κB signaling for transcriptional activation can occur through the classical (canonical) or the alternative (non-canonical) pathway, with both pathways leading to nuclear translocation and activation of NF-κB and binding of DNA. Both the canonical and non-canonical NF-κB pathways were found to be activated in IECs by IL-1β [24]. Interestingly, it was reported that hydroxylase inhibition led to the suppression of IL-1β-induced NF-kB-dependent gene expression [58]. Moreover, HIF-1α has been shown to repress the activation of NF-κB under inflammatory conditions [2]. In our experiments, LPS failed to induce an increase in HIF-1α levels; consistently, it has been demonstrated in a murine macrophage cell line that LPS stimulation induced the HIF-1α downregulation to below the basal level after an initial increase leading to pyroptosis [59]. Here, we demonstrated that SLAB51 lysate was able to counteract the NF-κB activation and IL-1β secretion induced by LPS stimulation. The probiotic also counteracted NOS2 expression and activity induced by LPS treatment. Notably, it has been shown that while acute exposure to NO stabilizes HIF-1α, chronic exposure to NO destabilizes HIF-1α [30].

SLAB51 possesses high levels of arginine deiminase (ADI) [33]. This prokaryotic enzyme, using the same substrate as NOS, L-arginine, indirectly inhibits the generation of NO due to substrate depletion [60]. The exposure of Caco-2 to SLAB51 induced a reduction in NOS2 activity [33]. In our conditions, the inhibition of the PI3K/AKT pathway was able to counteract the effect of SLAB51 on NOS activity, demonstrating a link between the activation of the AKT/PHD2/HIF pathway and the anti-inflammatory effect of the probiotic. Moreover, the inability of AKT signaling inhibition to totally counteract the effects of SLAB51 on NOS activity could be due to the ADI present in the probiotic.

In summary, our results broaden the knowledge of the beneficial effects of the multi-strain probiotic SLAB51, showing its ability to trigger a hypoxic response in intestinal epithelial cells in vitro under normoxic conditions. The increased HIF-1α levels associated with higher glycolytic metabolism and L-lactate production could be attributed to decreased PHD2 levels and activity, as well as to induced PI3K/AKT signaling. The ability of the probiotic lysate to activate the AKT pathway could be related to the modulation of TLR-mediated signaling. In this context, some probiotics or their bioactive compounds have been reported to modulate TLR signaling and inhibit the TLR4-mediated activation of inflammatory response elicited by LPS under inflammatory conditions [61,62,63].

It will be interesting to investigate the influence of SLAB51 treatment on the HIF pathway of the gut immune components under normoxia, physiological hypoxia, and pathological or inflammatory hypoxia. Furthermore, considering the suggested and partly opposite effects of HIF-1α vs. HIF-2α in gut homeostasis [11,64,65], it will be crucial to verify whether treatment with SLAB51 modulates the two isoforms of HIF differently. Identifying the SLAB51 lysate active components will also increase our understanding of the observed effect and advance the knowledge of evidence-based bacteriotherapy strategies.

Overall, these results support our hypothesis that, by increasing HIF-1α levels through the upregulation of the AKT pathway, the SLAB51 lysate can inhibit the NF-κB activation and its downstream signaling in IECs, counteracting the LPS-induced inflammatory response. To the best of our knowledge, this is the first report showing that a probiotic formulation led to HIF pathway activation in human IECs, by influencing PI3K/AKT signaling. In order to verify this hypothesis, studies are underway on supplementary cell lines and intestinal organoids using other complementary techniques and analyses, such as gene expression and flow cytometry. In addition, the suggestive hypothesis of potential immunomodulatory and anti-inflammatory bioactive peptides derived from SLAB51 strains will also be investigated in our experimental conditions [66,67].

Even though further studies are warranted to investigate the observed effects in further inflammatory models in vitro and in vivo, we can hypothesize that the SLAB51 formulation could act as a hypoxia mimetic agent, thus contributing to the preservation of intestinal homeostasis and the protection of IECs from the detrimental effects of chronic inflammation and pathologic hypoxia. On the other hand, a growing body of evidence supports that HIF-1α signaling can trigger contrasting responses depending on cell and tissue type [68]. Of particular interest, higher HIF-1α levels in diagnostic tumor biopsies have been associated with increased mortality risk in several cancers; these results were supported by experimental studies, which revealed that genetic manipulations leading to HIF-1α overexpression resulted in tumor overgrowth [69]. Conversely, the loss of HIF-1α activity resulted in reduced tumor expansion. Accordingly, HIF-1α overexpression has been observed in a variety of human cancers, including colon [70,71], and can enhance chemoresistance through the inhibition of apoptosis and senescence and the activation of drug efflux proteins [72]. Thus, targeting HIF-1α is considered a promising strategy for inhibiting cancer cell proliferation and overcoming chemoresistance. Of note, our preliminary and encouraging results indicate that the SLAB51 lysate was able to counteract the stabilization of HIF-1α in different human colorectal cancer cell lines, i.e., HT29 and HCT116 (not shown).

In conclusion, our data can partially explain the beneficial effects recorded following treatment with SLAB51 in animal models and patients with hypoxia-related conditions [31,32,33,34,35,36]. Despite this, to furnish the multi-strain SLAB51 probiotic with an authorized health claim approved by a regulatory agency, such as the U.S. Food and Drug Administration (FDA) or the European Food Safety Authority (EFSA), evidence has to be provided through well-designed human trials.

## 4. Materials and Methods

### 4.1. Preparation of Bacterial Lysate for Cell Treatments

SLAB51 probiotic formulation (sold as Sivomixx, Ormendes SA, Jouxtens-Mézery, Switzerland) contains eight different live bacterial strains: *Streptococcus thermophilus* DSM 32245, *Bifidobacterium lactis* DSM 32246, *Bifidobacterium lactis* DSM 32247, *Lactobacillus acidophilus* DSM 32241, *Lactobacillus helveticus* DSM 32242, *Lactobacillus paracasei* DSM 32243, *Lactobacillus plantarum* DSM 32244, and *Lactobacillus brevis* DSM 27961. Bacterial lysate was prepared as previously described [73]. Briefly, SLAB51 formulation was suspended at the concentration of 133 × 10^9^ CFU in 10 mL of phosphate buffered saline (PBS, Euro Clone, West York, UK), centrifuged at 8600× *g*, washed twice, and sonicated (30 min, alternating 10 s of sonication and 10 s of pause) using a Vibracell sonicator (Sonic and Materials, Danbury, CT, USA). Bacterial cell disruption was verified by measuring the absorbance of the sample at 590 nm with a spectrophotometer (Eppendorf Hamburg, Germany) before and after every sonication step. The samples were then centrifuged at 17,949× *g*, and the supernatants were filtered using a 0.22 µm-pore filter (Corning Incorporated, Corning, NY, USA) to remove any whole remaining bacteria. Total protein content was determined by a Bradford assay, using bovine serum albumin (BSA, Sigma Aldrich, St. Louis, MO, USA) as the standard.

### 4.2. Caco-2 IECs

The Caco-2 cell line purchased from Sigma-Aldrich (St. Louis, MO, USA) was grown in tissue culture flasks at 37 °C, 5% CO_2_, and 90% relative humidity environment. The culture medium (Dulbecco’s modified Eagle’s medium, DMEM), supplemented with 10% fetal calf serum (FCS), 1% non-essential amino acids, 1 mM sodium pyruvate, 2 mM L-glutamine, 100 U/mL penicillin, and 100 μg/mL streptomycin (Euro Clone, West York, UK), was refreshed every other day. After reaching 80% confluence, cells were detached with trypsin solution from bovine pancreas (Euro Clone, West York, UK) and seeded into Dickinson 96-well plates (Seahorse XF96 Cell Culture Microplate, Agilent, Santa Clara, CA, USA) for metabolic studies, or into sterile tissue culture 6-well plates (Becton, San Jose, CA, USA) for the other experiments. In both the culture plates, the cells were seeded at 60,000 cells/cm^2^ and cell growth was monitored by microscopy. Fourteen days post-confluence, cells were incubated with or without the SLAB51 lysate at the indicated concentrations and with or without 10 µg/mL lipopolysaccharide (LPS, Sigma-Aldrich). Where indicated, the cells were also pre-treated for 30 min with 100 µM NOS2 inhibitor N-(3-(aminomethyl) benzyl) acetamidine (1400W) (Sigma-Aldrich, St. Louis, MO, USA) prior to the treatment with LPS. To inhibit the PI3K/AKT pathway, the cells were pre-treated for 30 min with perifosine at 20 µM (AKT inhibitor, Cell Signaling Technology, Danvers, MA, USA) or LY294002 10 µM (PI3K inhibitor, Cell Signaling Technology). MG132 10 µM (Merck KGaA, Darmstadt, Germany) was used to inhibit proteasomal degradation of hydroxylated HIF-1α. The iron chelator deferoxamine (DFO, 150 µM) was used to inhibit PHD2 activity. To evaluate the effect of the several treatments on cell viability and proliferation, the cells were washed with PBS, centrifuged for 10 min at 400× *g*, and the pellets incubated with a 0.04% Trypan blue (Euro Clone, West York, UK) solution for 5 min to analyze cell number and viability. Untreated cells were also analyzed and served as controls. Cells were transferred to a Bürker counting chamber and counted by microscopy (Eclipse 50i, Nikon Corporation, Minato, Tokyo, Japan). All the experimental conditions and treatments did not significantly influence the cell viability (always >90%) or basal proliferation level compared to the untreated controls at all the incubation times.

### 4.3. Western Blot Analysis

Cells were washed with cold PBS and removed from plates by scraping in RIPA Lysis Buffer (Merck KGaA, Darmstadt, Germany) containing 100 mM protease inhibitor cocktail (Sigma-Aldrich, St. Louis, MO, USA). The process was carried out on ice because HIF-1α degrades rapidly in normoxia. After the lysis, the samples were centrifuged at 17,949× *g* to eliminate cell debris. The supernatants were collected and assayed for protein content with a Bradford assay/DC Protein Assay (Bio-Rad Laboratories, Hercules, CA, USA). Then, 25 µg total proteins were resolved on a 10% sodium dodecyl sulphate–polyacrylamide gel electrophoresis (SDS-PAGE), and electroblotted onto nitrocellulose membranes (Bio-Rad Laboratories). Membranes were blocked with 5% nonfat dry milk for 1 h at room temperature and then incubated overnight at 4 °C with rabbit monoclonal antibody anti-HIF-1α (Cell Signaling Technology, Danvers, MA, USA) 1:1000, rabbit monoclonal antibody anti-Hydroxy-HIF-1α (Pro564, Cell Signaling Technology) 1:1000, rabbit monoclonal antibody anti-PHD2 (Abcam, Cambridge, UK) 1:1000, rabbit monoclonal antibody anti-phospho-AKT (Ser473, Cell Signaling Technology) 1:1000, rabbit monoclonal antibody anti-AKT (Cell Signaling Technology) 1:1000, rabbit polyclonal anti-NOS2 antibody (Boster Biological Technology, Pleasanton, CA, USA) 1:500, rabbit monoclonal antibody anti-NF-kB (Cell Signaling Technology), or with mouse monoclonal antibody anti-β-actin 1:1000 (OriGene Technologies, Inc, Rockville, MD, USA). Horseradish peroxidase- (HRP-) conjugated goat anti-rabbit IgG secondary antibody (Millipore EMD, Darmstadt, Germany) 1:2000 or HRP-conjugated goat anti-mouse IgG secondary antibody (Bio-Rad Laboratories, Hercules, CA, USA) 1:2000 were used. Immunoreactive bands were visualized by enhanced chemiluminescence (ECL, Amersham Pharmacia Biotech) according to the manufacturer’s instructions. Band relative densities were determined using a chemiluminescence documentation system Alliance (UVITEC, Cambridge, UK), and values were given as relative units (Supplementary Figures S1–S3).

### 4.4. L-Lactate Assay

L-lactate levels in cell culture supernatants were analyzed via the L-lactate assay kit (Abcam, Cambridge, UK) according to the manufacturer’s instructions. Accordingly, the supernatants were deproteinized with a 10 kDa NMWCO Centrifugal Filter Unit (Amicon, Millipore, Burlington, MA, USA), and the filtrate was added to reaction wells. The absorbance was measured by spectrophotometric reading at 570 nm using a microplate reader (Bio-Rad Laboratories, Hercules, CA, USA). The L-lactate levels were determined by comparison to a standard curve.

### 4.5. Metabolic Studies

Cells treated as described above were assessed for extracellular acidification rate (ECAR) and O_2_ consumption rate (OCR) to calculate glycolysis rate (ECAR/OCR) using the Seahorse XFe96 Analyzer (Agilent, Santa Clara, CA, USA) following the manufacturer’s instructions. Briefly, on the day of the assay, the medium was changed for Seahorse XF DMEM Medium pH 7.4 supplemented with glucose (10 mmol/L), pyruvate (1 mmol/L), and glutamine (2 mmol/L) (Agilent), and the cells were allowed to equilibrate in a non-CO_2_ incubator for 1 h; OCR and ECAR were then measured. XFp Mito Stress Test Kit was used to test mitochondrial function. Injection of oligomycin (1 μM), carbonyl cyanide-4 (trifluoromethoxy) phenylhydrazone (FCCP, 1 μM), and the mixture of rotenone and antimycin A (1 μM) allows for the determination of the key bioenergetic parameters: basal respiration, ATP production-linked respiration (ATP production), maximal respiration, spare respiratory capacity, nonmitochondrial respiration, proton leak, and coupling efficiency.

### 4.6. IL-1β ELISA

The levels of released IL-1β were quantified in the cell supernatants using a human IL-1β enzyme-linked immunosorbent assay (ELISA) kit (Sigma Aldrich, Saint Louis, MO, USA), as described in the manufacturer’s instructions. The collected media were cleared of cellular debris/dead cells by centrifugation at 1000× *g* for 15 min. The absorbance was measured by spectrophotometric reading at 450 nm. The IL-1β concentration was determined by comparison to a standard curve. Results are expressed as pg/mL.

### 4.7. Nitrite Level Assay

The enzymatic activity of NOS2 was evaluated by measuring nitrite levels using nitrate reductase and Griess reaction through a colorimetric assay (Nitrite Assay kit-Sigma-Aldrich Co., Milan, Italy). Nitrite levels were assayed in the supernatants of Caco-2 cells, treated as above described, applied to a 96-well microtiter plate, according to the manufacturer’s instructions. The absorbance was measured by spectrophotometric reading at 550 nm using a microplate reader (Bio-Rad Laboratories). The nitrite content of each sample was evaluated with a standard curve obtained by linear regression made with sodium nitrite and expressed as fold vs. control.

### 4.8. Statistical Analysis

Statistical analysis of data was performed using GraphPad Prism 6.0 (GraphPad Software, San Diego, CA, USA). For comparison between two means, Student’s unpaired *t*-test was used. For comparison of the mean values among the groups, a one-way ANOVA, followed by Dunnett or Tukey post hoc test, was used. The results were expressed as mean ± SEM, as specified in figure legends. *p* values less than 0.05 were considered to be statistically significant.

## Data Availability

The datasets generated and analyzed during the current study are available from the corresponding authors upon reasonable request.

## Supplementary Materials

The following supporting information can be downloaded at: https://www.mdpi.com/article/10.3390/ijms24098134/s1.
