# Supplementary material for: Bacterial Lysate from the Multi-Strain Probiotic SLAB51 Triggers Adaptative Responses to Hypoxia in Human Caco-2 Intestinal Epithelial Cells under Normoxic Conditions and Attenuates LPS-Induced Inflammatory Response
Source: Int J Mol Sci. 2023 May 2;24(9):8134. doi: 10.3390/ijms24098134 (PMC10179068; doi:10.3390/ijms24098134)
Supplement: Supplementary file 1 [file ijms-24-08134-s001.zip › ijms-2189862-supplementary.pdf]

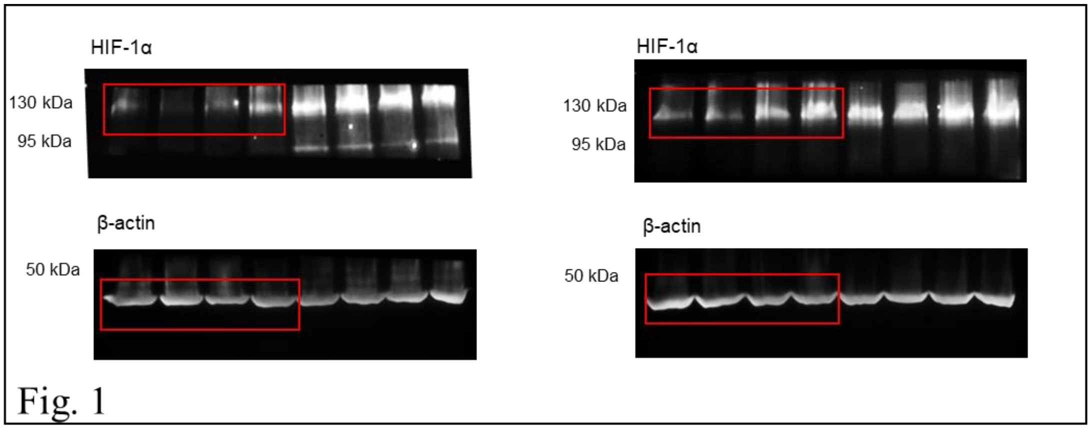

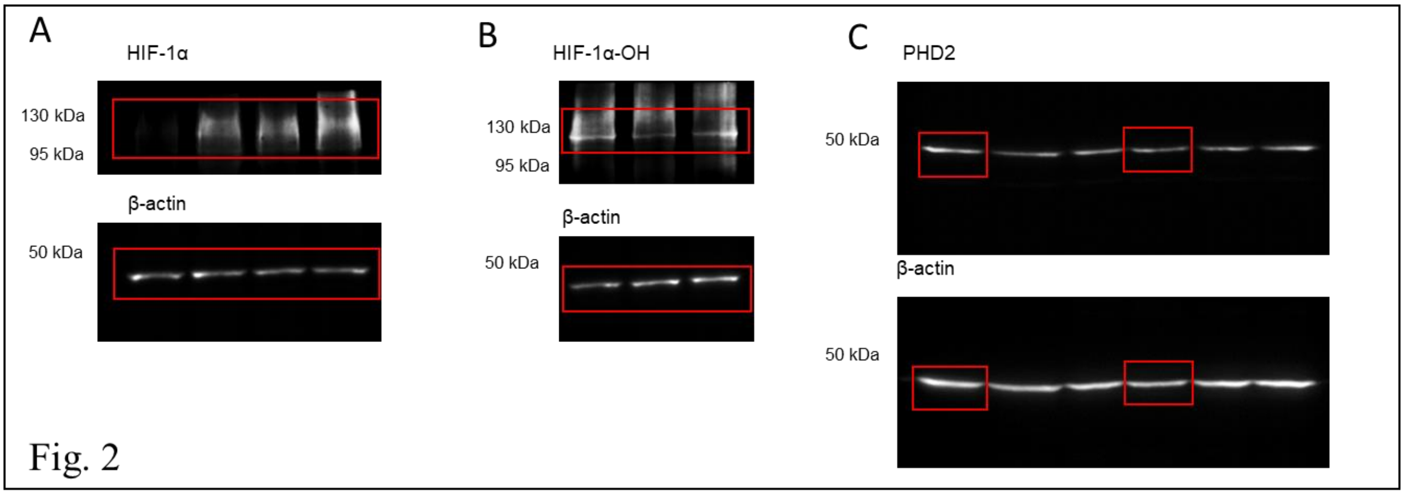

Supplementary Figure 1. Uncropped Western blot images from the blots shown in Figure 1 and 2. The red boxes outline the areas presented in the figures.

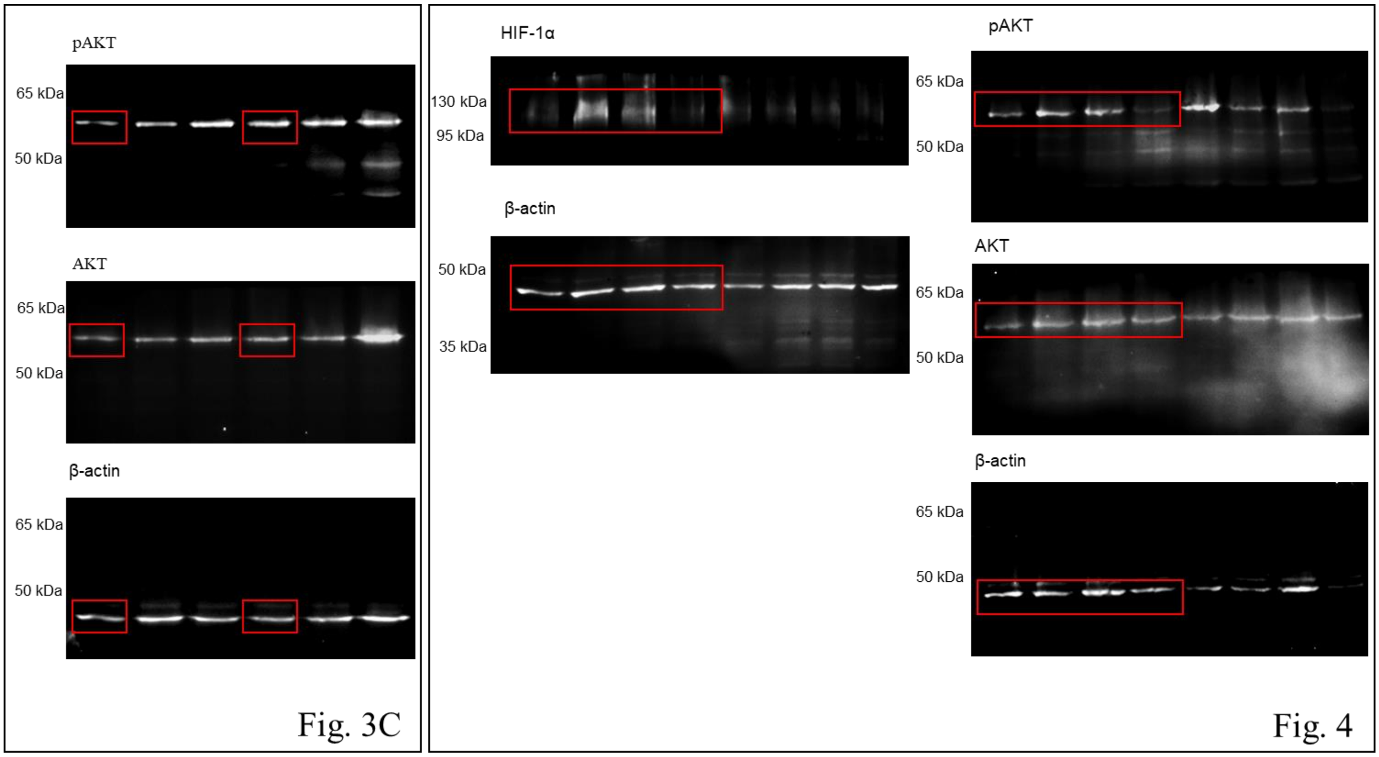

Supplementary Figure 2. Uncropped Western blot images from the blots shown in Figure 3 and 4. The red boxes outline the areas presented in the figures.

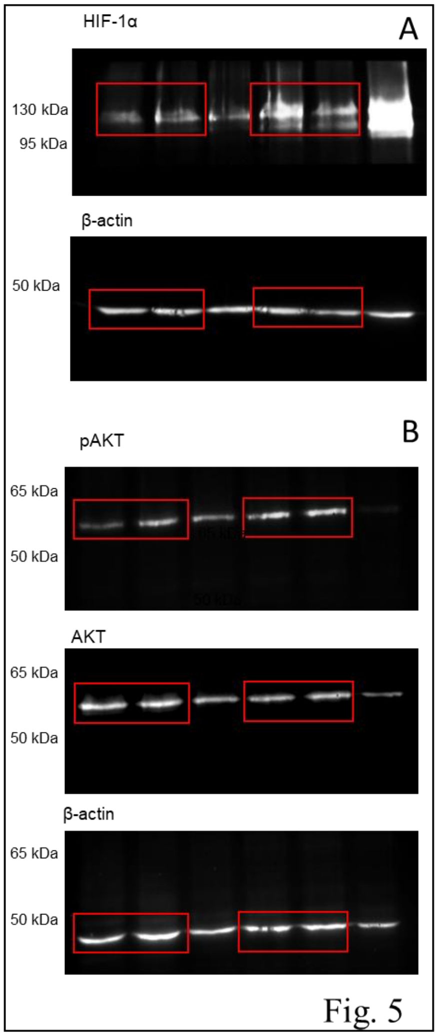

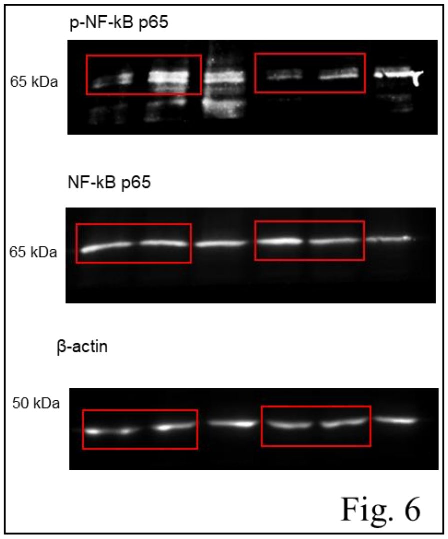

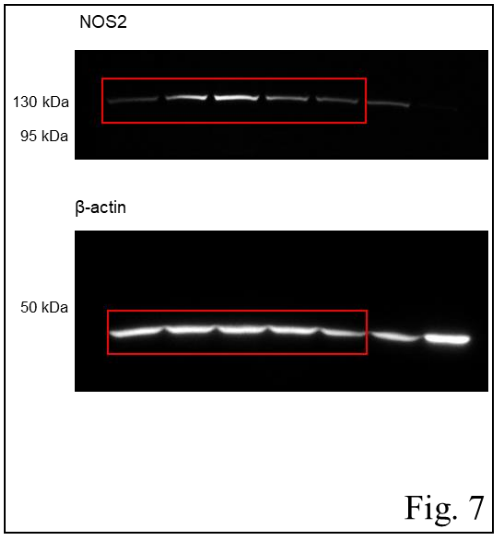

Supplementary Figure 3. Uncropped Western blot images from the blots shown in Figure 5, 6, and 7. The red boxes outline the areas presented in the figures.
